# The Hippoboscidae (Insecta: Diptera) from Madagascar, with new records from the “Parc National de Midongy Befotaka”

**DOI:** 10.1051/parasite/2011182127

**Published:** 2011-05-15

**Authors:** N. Rahola, S.M. Goodman, V. Robert

**Affiliations:** 1 MIVEGEC (Maladies Infectieuses et Vecteurs : Écologie, Génétique, Évolution et Contrôle), IRD 224, CNRS 5290, Université Montpellier 1, Université Montpellier 2 Montpellier France; 2 Field Museum of Natural History Chicago Illinois United States; 3 Vahatra Antananarivo Madagascar

**Keywords:** Hippoboscidae, birds, review, determination key, hostparasite relationship, taxonomy, Madagascar, Midongy Befotaka, Hippoboscidae, oiseaux, revue, clé de détermination, relations hôte-parasite, taxonomie, Madagascar, Midongy Befotaka

## Abstract

The Hippoboscidae or “louse-flies” is a family of pupiparous Diptera, which in their adult stage are ectoparasites of mammals and birds. This paper presents a comprehensive review of Malagasy Hippoboscidae. In total, amongst the 213 species of this family known worldwide, 14 have been reported in Madagascar, among which six are considered as endemic to the Malagasy region. In addition, data are presented from a collection of 17 Hippoboscidae obtained from seven species of forest-dwelling birds in the “Parc National de Midongy Befotaka”, southeastern Madagascar, in 2003. The flies in this collection belong to three different species: *Icosta malagasii* (one), *Ornithoica podicipis* (ten) and *Ornithoctona laticornis* (six). The two former species were previously only known from single specimens in museum collections; the later species is distributed across much of the Afrotropical region and the records presented herein are the first for Madagascar. All the seven bird species are new hosts for hippoboscids. We present the first description of the male of *Icosta malagasii*. An illustrated dichotomous determination key of the 14 Malagasy species, based on morphological criteria only, is presented.

## Abstract (fr)

Les Hippoboscidae constituent une famille de Diptères pupipares dont le stade adulte est ectoparasite de mammifères et d’oiseaux. Nous présentons ici une revue de tous les Hippoboscidae connus de Madagascar. Parmi les 213 espèces de cette famille connues dans le monde, 14 ont été observées au moins une fois à Madagascar, parmi lesquelles six sont considérées comme endémiques de la région malgache. Nous présentons aussi la récolte de 17 Hippoboscidae adultes collectés de sept espèces d’oiseaux strictement forestiers en 2003 dans le Parc National de Midongy Befotaka, sud-est de Madagascar. Ces 17 hippobosques se répartissent en trois espèces : *Icosta malagasii* (un), *Ornithoica podicipis* (dix) et *Ornithoctona laticornis* (six). Les deux premières de ces espèces étaient précédemment connues par un unique spécimen enregistré dans les collections des muséums ; *O. laticornis* a une large répartition en zone Afrotropicale, mais il est trouvé pour la première fois à Madagascar. Chacune des sept espèces d’oiseaux constitue un nouvel hôte pour les hippobosques collectés. Nous proposons la première description du mâle d’*Icosta malagasii*. Nous présentons enfin une clé dichotomique illustrée, basée exclusivement sur des critères morphologiques, pour la détermination des 14 espèces malgaches.

## Introduction

The members of the family Hippoboscidae, known as “louse flies” or “keds”, are obligate ectoparasites of mammals and birds. Members of this Diptera family consist of winged species, some of which have considerable flight capacity, while other species are flightless and highly apomorphic, having vestigial or no wings. Hippoboscidae belong to Pupipara “pupa-bearers”, in reference to the fact that, unlike virtually all other insects, most of the larval development takes place within the mother’s abdomen and pupation occurs almost immediately after pre-pupae laying (Hutson, 1984). The Hippoboscidae belongs to the super-family of Hippoboscoidea, which groups four families of flies that are bloodsucking at the adult stage for both sexes. The four families consist of Hippoboscidae, the bat flies Nycteribiidae and Streblidae, and the tsetse flies Glossinidae. Hippoboscoidea are considered monophyletic, but based on current data, the basal relationships between the four families are not resolved (Dittmar *et al.*, 2006). A recent checklist of Hippoboscidae across the world retains three subfamilies (Ornithomyinae, Hippoboscinae, and Lipopteninae), 21 genera, and 213 species (Dick, 2006). It has been shown that the two subfamilies Hippoboscinae and Lipopteninae are monophyletic groups (Petersen *et al.*, 2007).

Caution must be taken with the name Hippoboscidae. In old taxonomic treatments, it was used to encompass the Hippoboscidae as defined today, as well as the bat-flies (Nycteribiidae and Streblidae). Irwin *et al.* (2003), following the BDWD (BioSystematic Database of World Diptera), used this old definition and indicated 760 valid species in the world and 15 in Madagascar, among which seven are Hippoboscidae *sensu stricto*.

Members of the Hippoboscidae are known to act as vectors of many infectious agents: protozoan, bacteria, helminthes, and perhaps viruses. They certainly transmit mammal Trypanosomatidae of the genus *Megatrypanum* (Baker, 1967; Oyieke & Reid, 2003) and probably transmit avian trypanosomes (Kucera, 1983). Hippoboscidae are the only known vectors of *Haemoproteus* (an apicomplexan parasite of birds). *Melophagus ovinus* louse flies play a role in the transmission of *Bartonella* among ruminants (Halos *et al.*, 2004; Reeves *et al.*, 2006). In Kenya, the fly *Hippobosca longipennis* is thought to transmit to hyenas and domestic dogs the larva of the filarial nematoda *Acanthocheilonema dracunculoides* (Nelson, 1963). This filaria has been reported from domestic animals in Madagascar (Daynes, 1964). The louse fly *Icosta americana* is currently suspected in the transmission of West Nile Virus in North America (Farajollahi *et al.*, 2005).

Although the importance of Hippoboscidae in the transmission of mammal and bird parasites in Madagascar has been suspected and to some extent documented (Daynes, 1964; Savage *et al.*, 2009), members of this family remain poorly documented on the island. We here present the first comprehensive review of Malagasy Hippoboscidae (*sensu stricto i.e.* excluding Nycteribiidae and Streblidae). In addition, we propose the first illustrated dichotomous determination key for the genera and species currently known from Madagascar. Recent specimens of hippoboscids have been collected from Malagasy vertebrates and remain unstudied by fly specialists (*e.g.*, Wright *et al.*, 2009). Herein we present details on a hippoboscid collection obtained from forest-dwelling birds in the “Parc National de Midongy Befotaka”, previously known as Midongy-Sud, southeastern Madagascar.

## Review of Malagasy Hippoboscidae Based on Published Literature

Information on Malagasy Hippoboscidae is rather limited and notably dispersed in the scientific literature. We found published records of 13 species recorded at least once on Madagascar. The host species (when known) and/or the context for Madagascar are presented in Table I. The numbers of species from the three subfamilies of Hippoboscidae – Ornithomyinae, Hippoboscinae, and Lipopteninae – are 11, two, and zero, respectively. Six species of Ornithomyinae are presently considered as endemic to Madagascar. Individuals of eight species have been collected from a single individual host, including three different specific names proposed for the female flies (*Icosta malagasii*, *Ornithomya sorbens*, and *Ornithoctona idonea*), while the identity of the male remains unknown.

**Table I.** Hippoboscidae recorded at least once on Madagascar based on information from the literature.

Subfamily	Species	Hosts	Distribution	Main references	Context for Madagascar
Ornithomyinae	*Allobosca crassipes* Speiser, 1899	Mammals, lemur (Lemuridae and Indriidae)	Madagascar	Ferris & Cole, 1922; Maa, 1969c; Vaughn and Mcgee, 2009	Known from seven lemur species ex. *Eulemur macaco, E. rubriventer, Lepilemur mustelinus, Avahi laniger, Varecia variegata variegata, Propithecus diadema and P. edwardst*
	
	*Proparabosca alata* Theodor & Oldroyd, 1965	Mammals, lemurs (Indriidae)	Madagascar	Theodor & Oldroyd, 1965	One record (1 ♂ + 1 ♀); known from one lemur species ex Propithecus *verreauxi* coronatus from Namoroka
	
	*Icosta ardeae ardeae* (Macquart, 1835)	Birds, Ardeidae	Cosmopolitan except Americas	Maa, 1969b	Similar to continental Africa
	
	*Icosta minor* (Bigot, 1858)	Birds, Passeriformes and Cuculidae	Africa, Mediterranean Bassin	Maa, 1964	One record (1 ♀) ex *Centropus toulou* from Marovoay
	
	*Icosta malagasii* Maa, 1969	Unknown birds, probably Falconiformes	Madagascar, perhaps Grande Comore	Maa, 1969b	One single female “parasite sur l’aile d’un Coezach” (see text) from Sahafanjana, ♂ previously unknown
	
	*Ornithoctona idonea* Falcoz, 1929	Birds, Leptosomatidae	Madagascar	Falcoz, 1929; Maa, 1969a	One record (1 ♂ + 2 ♀) ex *Leptosomus discolor*, no precise locality, no material available, $ not described
	
	*Ornithoctona plicata* von Olfers, 1816	Birds (12 orders of birds)	Cosmopolitan except Americas	Maa, 1969c	Similar to continental Africa
	
	*Ornithoica podicipis* von Röder, 1892	Birds, Ardeidae	Africa	Bequaert, 1954; Maa, 1966	One uncertain record ex Bubulcus *ibis*, no precise locality, no precise sex
	
	*Ornithoica hovana* Maa, 1966	Birds, Threskiornithidae	Madagascar	Maa, 1966	One record (1 ♂, 3 ♀) ex *Lophotibis cristata*, from Tanosy, Tolagnaro
	
	*Ornithomya sorbens* Hutson, 1971	Birds, Hirundinidae	Madagascar	Hutson, 1971	One record (1 ♀) ex unknown swallow species, from “km 50 route Majunga”, ♂ unknown
	
	*Pseudolynchia canariensis* (Macquart, 1840)	Wild birds and domestic pigeons	Cosmopolitan	Maa, 1962	One record (1 ♂) from Maroantsetra, Tamatave, host not indicated, probably domestic pigeon

Hippoboscinae	*Hippobosca variegate* Megerle, 1803	Big domestic mammals, horses and cattle	Sub-Saharan Africa and Asia	Maa, 1962	A lot of records, localities and vertebrate hosts are indicated
	
	*Hippobosca longipennis* Fabricius, 1805	Mammals, Carnivora (Canidae, Hyaenidae, Viveridae, Felidae)	Africa, Europe, Asia	Maa, 1969c	A lot of records, localities and vertebrate hosts are indicated

The genus *Allobosca* is composed by only one known species, *A. crassipes*. This species, considered endemic to Madagascar, has rudimentary wings and is known from several species of lemurs (Ferris & Cole, 1922; Maa, 1969c). *Proparabosca* is a genus of Hippoboscidae, with only one known species, *P. alata*, which is an ectoparasite of the Indriidae *Propithecus verreauxi* in Namoroka National Park (Theodor & Oldroyd, 1965). Most of the 171 species of Ornithomyinae are bird ectoparasites, with the exceptions of *A. crassipes* and *P. alata* (plus five species that parasite wallabies in Australia).

*Icosta malagasii* is only known by one female. It was collected as a “parasite sur l’aile d’un Coezach/ Sahafanjana/N: 115/R. M./ Inst. Sci. Madagascar” (Maa, 1969b). Sahafanjana is located in close proximity to the “Parc National de Mananara-Nord” and in the zone between Manambato and Anove (Quentin & Villiers, 1973; Flint *et al.*, 1987). One possibility in the identification of the “Coezach” is that it is a poor transliteration of the Malagasy vernacular name “Koa”, which are members of the endemic genus *Coua* (subfamily Couinae). In certain Malagasy dialects, the last syllable of the vernacular name is accented and harshly pronounced. In the Parc Botanique et Zoologique de Tsimbazaza, Antananarivo (previously known as “Institut de Recherche Scientifique de Madagascar”) there is a male specimen of *C. caerulea*, collected on 12 April 1955 by “R.M.” at Sahafanjana. This information gives credence to the identity of the “Coezach” as a *Coua*.

*Ornithoctona idonea* is only known by one male and two females with two of these specimens, including the male holotype, being from Madagascar (Falcoz, 1929). Theses two Malagasy specimens formerly in the Falcoz collection are now untraceable. Curiously, although the male was designated as the holotype, it remains undescribed. Presently, the only available specimen is a pined female *O. idonea* (in the Muséum national d’Histoire naturelle, Paris), collected in “Africa” (no precise locality, no host record) and designed as a paratype by Falcoz (1929). Maa (1963) suppressed this species as a synonym of *Ornithoctona laticornis*, but subsequently (1969a) retained *O. idonea* as a dubious species waiting for the rediscovery of the holotype or the availability of new material from Madagascar.

*Pseudolynchia canariensis*, the pigeon louse fly, is found in most parts of the tropical and sub-tropical areas of the world and associated with domestic pigeons and doves. *Pseudolynchia canariensis* is the vector for the Apicomplexa protozoan *Haemoproteus columbae*, a parasite that can be fatal to young pigeons.

*Hippobosca variegata*, a species distributed in sub- Saharan Africa and Asia, has also been observed in the Comoros Archipelago, specifically on Grande Comore and Mayotte (Brunhes, 1978). The natural host of this fly is unknown (Maa, 1969c). This species is common on Malagasy cattle (Maa, 1962). *Hippobosca longipennis* is known from introduced carnivorans on Madagascar (Bequaert, 1939).

On the Aldabra Atoll, Seychelles, which is 420 km NW of Madagascar, Cogan *et al.* (1971) reported that local land birds were rarely and only lightly parasitized with one unspecified species of Hippoboscidae. In contrast, bird species occurring in marine environments had notably higher concentrations of parasites, particularly far-ranging groups like the boobies and tropicbirds heavily parasitized by *Olfersia aenescens* and frigates by *Olfersia spinifera*.

## Original Observations on Hippoboscidae from the “PARC National De Midongy Befotaka”

### Study Area

The “Parc National de Midongy Befotka” is located in the southeastern portion of Madagascar and along the western flank of a chain of northsouth oriented mountains running the length of much of eastern Madagascar. All of the sites visited were in undisturbed or largely undisturbed natural forest classified as “Humid Forest” (Moat & Smith, 2007). Meteorological patterns in the village of Midongy-Sud, in close proximity to the park, include an average of 1,825 mm of rain per year, falling on 170 days, with the wettest month being January, and the annual mean temperature of 18 °C (Donque, 1975). Hence, the zone does not have a pronounced dry season.

### Biological Material

During field expeditions, ornithologists mist-netted birds in the “Parc National de Midongy Befotaka”. Four-panelled mist-nets measuring 12 × 2.4 m with 36 mm mesh were installed at ground level in the forest understory. After being dispatched, certain birds retained as voucher specimens and for other research purposes, were individually placed in clean plastic bags with a small cotton ball moisten with ethyl acetate. All ectoparasites recovered were preserved in ETOH and are held in the collections of the Field Museum of Natural History, Chicago.

All the Hippoboscidae reported on herein were collected from birds captured between 27 October and 14 November 2003; other details on the hosts are presented in Table II.

**Table II.** Hippoboscidae from the “Parc National de Midongy Befotaka”, Madagascar, and characteristics of their bird hosts. Specimens held in the Field Museum of Natural History (FMNH) and the Université d’Antananarivo, Département de Biologie Animale (UADBA). Specimens in the UADBA collection have yet to be catalogued and are referred to by the collection number.

Determination of genus based on morphological criteria	Determination of species group based on morphological criteria	Determination of species (see text)	Number and sex of hippoboscids per bird host	Bird host	Catalogue number of host	Field catalogue of collectors’^d^	Collection date (in 2003)	Collection longitude	Collection latitude	Collection altitude (m)
*Icosta* Speiser, 1905	*plana*	*Icosta malagasii* Maa, 1969	1 ♂	*Accipiter francesii* Smith, 1834^a^	UADBA DW 5579	DW 5579	14 Nov	47°03.1’E	23°30.6’S	650

*Ornithoctona* Speiser, 1902	*australasiae*	*Ornithoctona laticornis* (Macquart, 1935)	1 ♀	*Atelornis crossleyi* Sharpe, 1875^b^	FMNH 438665	SMG 13910	3 Nov	46°57.5’E	23°50.3’S	1,250
			1 ♂ + 2 ♀	*Atelornis pittoides* (Lafresnaye, 1834)^b^	FMNH 438661	DW 5514	3 Nov	46°57.5’E	23°50.3’S	1,250
			1 ♀	*Atelornis pittoides* (Lafresnaye, 1834)	FMNH 438662	DW 5518	4 Nov	46°57.5’E	23°50.3’S	1,250
			1 ♀	*Monticola sharpie* (Gray, 1871)^b^	FMNH 438738	DW 5528	5 Nov	46°57.5’E	23°50.3’S	1,250

*Ornitboica* Rondani, 1878	*turdi*	*Ornitboica podicipis* von Röder, 1892	1 ♂	*Atelornis pittoides* (Lafresnaye, 1834)	FMNH 438660	DW 5458	27 Oct	46°57.8’E	23°50.1’S	875
			1 ♂ + 1 ♀	*Atelornis pittoides* (Lafresnaye, 1834)	FMNH 438662	DW 5518	4 Nov	46°57.5’E	23°50.3’S	1,250
			1 ♂ + 1 ♀	*Accipiter francesii* Smith, 1834	UADBA DW 5531	DW 5531	13 Nov	46°57.5’E	23°50.3’S	1,250
			1 ♂	*Accipiter francesii* Smith, 1834	UADBA DW 5543	DW 5543	6 Nov	46°57.5’E	23°50.3’S	1,250
			1 ♀	*Otus rutilus* (Pucheran, 1849)^b^, ^c^	UADBA DW 5568	DW 5568	13 Nov	47°03.1’E	23°30.6’S	650
			1 ♂ + 1 ♀	*Brachypteracias leptosomus* (Lesson, 1832)^b^	FMNH 438658	DW 5490	30 Oct	46°57.8’E	23°50.1’S	875
			1 ♀	*Streptopelia picturata* (Temminck, 1813)^a^	FMNH 438653	SMG 13944	7 Nov	46°57.5’E	23°50.3’S	1,250

Total			17 (10 ♀, 7 ♂)	11 birds belonging to seven bird species						

a Malagasy region endemic (Madagascar and the Comoros);

b Madagascar endemic;

c Following Fuchs *et al.* (2008) this species is endemic to Madagascar;

d DW = Dave Willard; SMG = Steven M Goodman.

The determination of these Hippoboscidae flies used Maa’s (1963) key at the levels of subfamily and genus. The species determination was subsequently inferred using other references (Maa, 1966 for *Ornithoica*; Maa, 1969a for *Ornithoctona*; and Maa, 1969b for *Icosta*). Following Maa (1969b), *O. idonea* “would be separable from *O. laticornis* in having antennal appendage relatively more acute at apex, palpus longer, mesosternal processes closer to each other and posterior marginal bristles on laterite two much shorter.”

### Results

In total, 17 Hippoboscidae specimens were collected from 11 individual birds belonging to seven different bird species (Table II). They belong to three species of Ornithomyinae, namely *Icosta malagasii*, *Ornithoica podicipis*, and *Ornithoctona laticornis*. The two former species were previously only known from single museum specimens and are here represented in the Midongy Befotaka collection by an additional one and ten individuals, respectively. Further, based on this material, the male of *I. malagasii* can now be described (see below) and for the first time *O. laticornis* is documented on Madagascar.

New records include collection of *I. malagasii* had been collected from *Accipiter francesii*; *Ornithoica podicipis* from *Atelornis pittoides*, *Accipiter francesii*, *Otus rutilus*, *Brachypteracias leptosomus*, and *Streptopelia picturata*; and *O. laticornis* from *Atelornis crossleyi*, *Atelornis pittoides*, and *Monticola sharpei*.

### Discussion

The specimens recorded from the “Parc National de Midongy Befotaka” are the first reported in detail from wild Malagasy forest-dwelling birds. Within this material, we identified specimens (five females and one male) as *O. laticornis* because they perfectly match Maa’s description (1969a). However, it is hard to definitely exclude *O. idonea* because of Falcoz’s imprecise drawings and description. Within these six specimens, there is notable polymorphism in antenna shape, setae of the first sternite, and ocellus area. Individuals of both sexes of *O. laticornis* in Madagascar support Maa’s (1963) suggestion that *O. idonea* is a synonym of *O. laticornis*. We present the second record of *O. podicipis* on Madagascar (following the first record used in the original description), with the identification of five new host birds for this hippoboscid. Given the high level of endemicity of Malagasy birds at different taxonomic levels, including 51% of the 209 nesting bird species (Goodman & Hawkins, 2008), it is rather striking that there has not been a greater radiation of hippoboscid species, as is known in other groups of blood-sucking flies parasites of vertebrates (*e.g.* Dick, 2007). Perhaps current low-levels of measured species diversity within Malagasy hippoboscids are in part associated with few field collections of these flies. Another explanation is that within the species groups with broad extralimital distributions, such as different genera within the Ornithomyinae, unrecognized cryptic species occur on Madagascar. Molecular genetic studies of recent collections of hippoboscids from Madagascar and neighbouring islands should help resolve these questions.

### Description of the Male of *Icosta (Icosta) Malagasii* Maa, 1969

**Plate 1.**
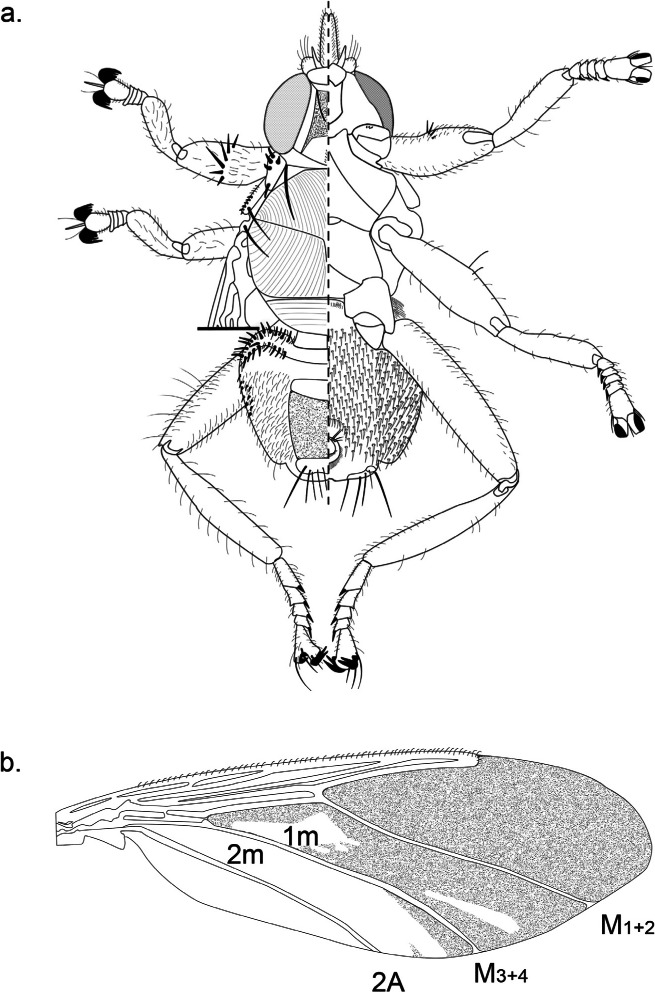
a. *Icosta (Icosta) malagasii*, ♂ in dorsal (left) and ventral (right) view; b. *Icosta (Icosta) malagasii*, ♂ wing.

The male is very similar to the female, as described by Maa (1969b), but differs as follows. Abdomen with three tergal plates, the first one is dark, narrow, lining the abdomen basally, and extending antero-laterally. The second tergal plate is linked to the third one by a broad striate zone bearing small and narrow setae. The third tergal plate (tergite VI) bears five to seven pairs of long dark bristles projecting posteriorly. Ventrally the urogenital area is surrounded by small and narrow setae and anteriorly by a spot of dark bristles. The antenna is notably less hirsute than in the female (Plate 1, Figs a, b).

## Dichotomic Key for Determination of Malagasy Hippoboscidae

• Key to Genera of Hippoboscidae

**Plate 2.**
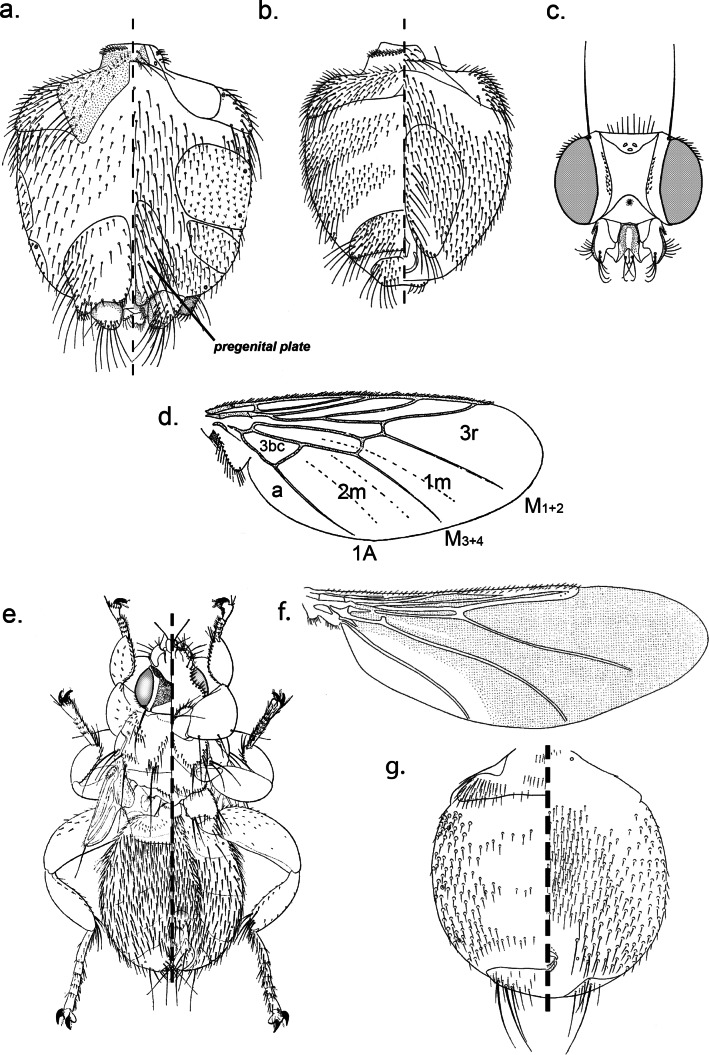
a. *Proparabosca alata*, ♀ abdomen in dorsal (left) and ventral (right) view; b. *Proparabosca alata*, ♂ abdomen in dorsal (left) and ventral (right) view; c. *Proparabosca alata*, head in dorsal view; d. *Proparabosca alata*, wing; e. *Allobosca crassipes*, ♀ in dorsal (left) and ventral (right) view; f. *Icosta (Ornithoponus) minor*, ♀ wing; g. *Icosta (Ornithoponus) minor*, ♀ abdomen in dorsal (left) and ventral (right) view.

1- Wing abnormal in size, frons longer than wide, hind femur almost as long as thick and fore tarsus with spatulate setae (Plate 2, Fig. e) ................... *Allobosca* (with one species of this genus recorded in Madagascar: *A. crassipes* Speiser, 1899)

- Wing fully developed and functional .................... 2

**Plate 3.**
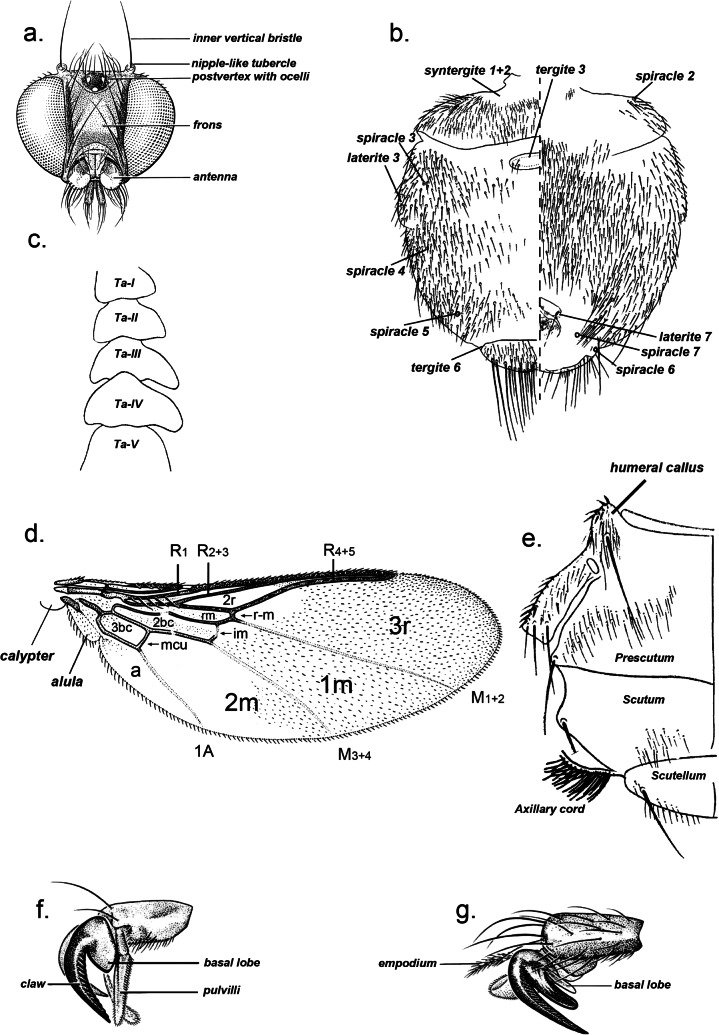
a. *Ornithoica* sp. head; b. *Icosta* sp. abdomen showing plates and spiracles in dorsal (left) and ventral (right) view; c. *Icosta* sp. tarsus I with the fives tarsomeres (Ta-III and Ta-IV are asymmetrical); d. *Ornithoica* sp. wing showing venation and cells (3bc, anal cell; a, axillary lobe; A, anal vein; R, radial; M, media; r-m, discal; im, postical; mcu, base of upper postical); e. *Icosta* sp. thorax in dorsal (left) view; f. tarsal claw simple; g. tarsal claw bidentate.

2- Wing (Plate 3, Fig. d) with 2 or 1 crossveins (*r-m*, *im*) ............................................................................... 3

- Wing with 3 crossveins (*r-m*, *im*, *mcu*). Ocelli present ...................................................................................... 5

3- Wing with only *r-m* crossvein present ..................... ................................................................. *Pseudolynchia* (with one species of this genus recorded in Madagascar: *P. canariensis* (Maquart, 1840))

- Wing with two crossveins ....................................... 4

4- Wing with 2 delineated crossveins, wing membrane of all open cells distinctly wrinkled. Pronotum large and forming an observable neck-like segment between head and mesonotum. Ocelli always absent ...... *Hippobosca*

**Plate 4.**
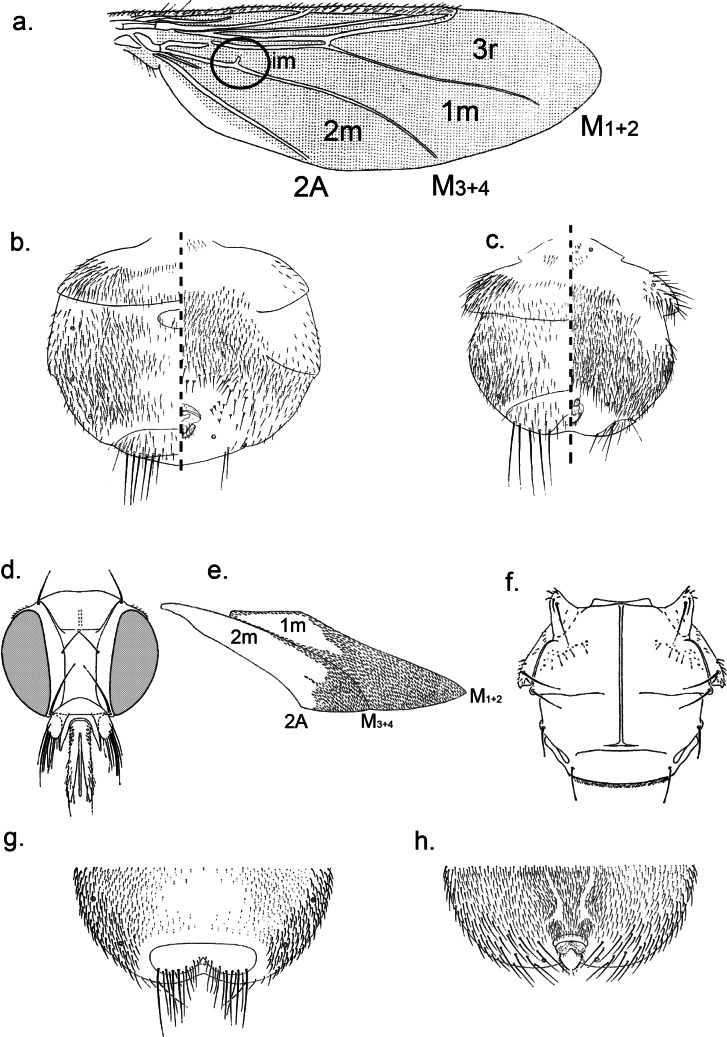
a. *Icosta (Ardmoeca) ardeae ardeae*, wing (A, anal vein; R, radial; M, media; im, postical); b. *Icosta (Ardmoeca) ardeae ardeae*, ♀ abdomen in dorsal (left) and ventral (right) view; c. *Icosta (Ardmoeca) ardeae ardeae*, ♂ abdomen in dorsal (left) and ventral (right) view; d. *Icosta (Icosta) malagasii*, ♀ head in front view; e. *Icosta (Icosta) malagasii*, ♀ wing (media cells only) with setulae distribution; f. *Icosta (Icosta) malagasii*, ♀ thorax in dorsal view; g. *Icosta (Icosta) malagasii*, ♀ abdominal apex in dorsal view; h. *Icosta (Icosta) malagasii*, ♀ abdominal apex in ventral view.

- Crossvein *im* with a transparent whitish spot (Plate 4, Fig. a) .................................................................. *Icosta*

5- Tarsal claws simple but seemingly bifid (because of the presence of a basal lobe which may be mistaken as basal denticle of claw) (Plate 3, Fig. f) ............... 6

- Tarsal claws bidentate but seemingly trifid (Plate 3, Fig. g) .......................................................................... 7

6- Wing densely ciliate along apical and anal margins and setulose at apical area. Vertical bristles each arising from nipple-like tubercle (Plate 3, Fig. a). Humeral callus (Plate 3, Fig. e) hardly protruding laterocephalad. Male hind trochanter with spine-like setae ...................................................................... *Ornithoica*

- Humeral callus weak, rounded not prominent with short setae at apex and 3 longer setae. Scutellum rhomboid without long setae. Wing with seven longitudinal veins. Anal cell strongly sclerotised and triangular. *M*_*1+2*_, *M*_*3+4*_ and anal vein ending a short distance before wing margin (Plate 2, Fig. d). Long pregenital plates on female abdomen (Plate 2, Fig. a) ...... *Proparabosca* (with one species of this genus recorded in Madagascar: *P. alata* Theodor & Oldroyd, 1965)

7- Wing with vein *2A* hardly visible and lower calypter exceptionally large. Antenna large, broad, leaf-shaped or spoon-shaped with distinct outer dorsal rim. Humeral callus very strong ................... *Ornithoctona*

**Plate 5.**
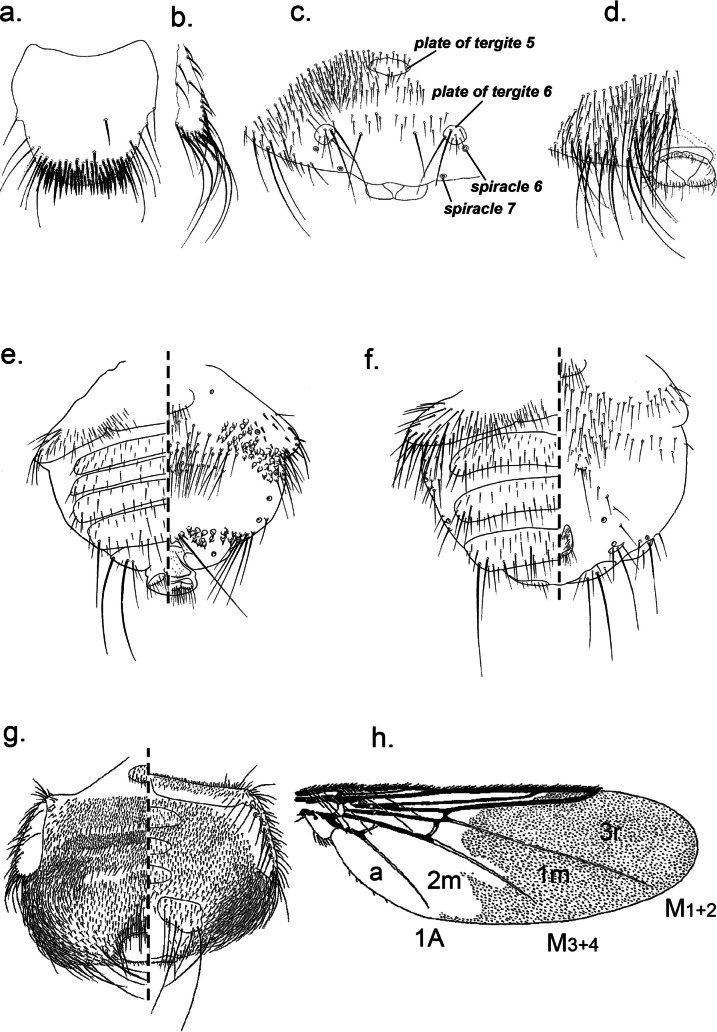
a. *Ornithoctona laticornis*, ♀ basal abdominal tergite; b. *Ornithoctona laticornis*, ♀ antero-lateral area of abdomen; c. *Ornithoctona laticornis*, ♀ abdominal apex in dorsal view; d. *Ornithoctona laticornis*, ♀ abdominal apex in ventral view; e. *Ornithoica (Ornithoica) hovana*, ♀ abdomen in dorsal (left) and ventral (right) view; f. *Ornithoica (Ornithoica) hovana*, ♂ abdomen in dorsal (left) and ventral (right) view; g. *Ornithomya sorbens*, ♀ abdomen in dorsal (left) and ventral (right) view; h. *Ornithomya sorbens*, ♀ wing (a, anal cell; A, anal vein; r, radial; m, media).

- Antenna small, narrow, never leaf- or spoon-like. Abdomen always with median tergal plates. Axillary cord fringed with pale soft hair ............... *Ornithomya* (with one species of this genus recorded in Madagascar: *O. sorbens* Hutson, 1971; Plate 5, Figs g, h)

• Key to species of the genus *Hippobosca* Linnaeus, 1758

1- Apical abscissa of vein *R*_*4+5*_, 4 to 5 times as long as full length of *R*_*2+3*_. Abdomen lacking median tergal plates.

*Hippobosca variegata* Megerle, 1803

- Apical abscissa of vein *R*_*4+5*_ not longer than full length of *R*_*2+3*_. Abdomen with 3 median tergal plates. Prosternum hardly shorter than wide, scutellum usually almost entirely pale.

*Hippobosca longipennis* Fabricius, 1805

• Key to species of the genus *Icosta* Speiser, 1905

1- Venter of femur III densely uniformly setose except an oval bare area at base. Male lacking tergite 3. Female with setae on median area of abdominal venter distinctly paler, longer and finer than those on submedian area which are more or less spine-like and similar to ventral setae near laterite 3 (Plate 4, Figs a, b, c).

*Icosta (Ardmoeca) ardeae ardeae* (Macquart, 1835)

- Venter of all femora bare besides few marginal setae ...................................................................................... 2

2- Tarsomeres 3 and 4 of foreleg clearly asymmetrical, with posterior apical lobes markedly longer than corresponding anterior ones (Plate 3, Fig. c); female urogenital area anteriorly fenced by pair of small setal patches. Wing setulae confined to cell *1m* et *2m*, cell *1m* fully covered except a basal bare spot very close to *M*_*3+4*_; cell *2m* almost entirely bare except at apex where setulae projecting anterally in a sharp process (Plate 4, Figs d, e, f, g, h).

*Icosta (Icosta) malagasii* Maa, 1969

- Tarsus 1 not apically asymmetrical. Lateral fence of ♀ urogenital area composed of 5 ± robust setae which are hardly longer than width of infra anal plate. Setae around ♂ abdominal spiracles 3-5 almost uniformly fine. Mesosternal process hardly visible. Metabasisternum never produced into posterolateral process (Plate 2, Figs f, g).

*Icosta (Ornithoponus) minor* Bigot, 1858

• Key to species of the genus *Ornithoctona* Speiser, 1902

1- Wing membrane entirely bare, at most with a very narrow setulose line on costal margin near apex; anterior mesosternal process distinctly shorter than interdistance of their apices; posterior ocelli hardly farther from each other than from anterior ocellus. Female abdomen lacking median tergal plates. Wing not less than 7 mm long.

*Ornithoctona plicata* von Olfers, 1816

- Wing membrane setulose ........................................ 2

2- Wing setulae covering part of cells *3r* and *1m* sometimes even apex of *2r* as well. Anterior mesosternal process narrow, distinctly longer than wide at base. Anterior ocellus situated on or slightly above level of posterior eye-margins. Female abdomen with 3 median tergal plates. Wing not more than 7.5 mm long (Plate 5, Figs a, b, c, d).

*Ornithoctona laticornis* (Macquart, 1835)

**Plate 6.**
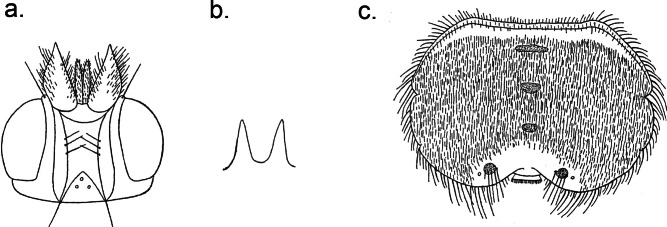
(from Falcoz’s 1929 drawings). a. *Ornithoctona idonea*, ♀ head; b. *Ornithoctona idonea*, ♀ mesosternal process; c. *Ornithoctona idonea*, ♀ abdomen in dorsal view.

- Wings partially setulose. Para-anal laterite bearing 3 to 4 long setae. First sternite rectangular and narrow with many rows of spine-like setae of unequal length on the posterior edge. Ocelli area equilateral triangle shaped. Female abdomen with 3 median tergal plates (Plate 6, Figs a, b, c).

*Ornithoctona idonea* Falcoz, 1929

• Key to species of the genus *Ornithoica* Rondani, 1878

1- Wing size 3.2-3.5 mm. Wing-setulae covering more than 1/2 of cell *2m* and extending to apical 1/2 or more of *2r* and to apical margin of *r-m*. Mesoscutum except small areas at its anterolateral corners, as densely setose as prescutum and scutellum. Bare area at base of cell *3r* much less than 2 × as that of *1m*.

*Ornithoica (Ornithoica) podicipis* von Röder, 1892

- Wing size 2.5-2.8 mm. Wing-setulae covering much less than 1/2 of cell *2m*, generally forming very small patch near its antero-apical corner; cell *2r* at most setulose at extreme apex. Cell *r-m* entirely bare, setulose area in cell *2m* small confined to antero-apical corner (Plate 5, Figs e, f).

*Ornithoica (Ornithoica) hovana* Maa, 1966.
